# High-Pitch CT Pulmonary Angiography in Third Generation Dual-Source CT: Image Quality in an Unselected Patient Population

**DOI:** 10.1371/journal.pone.0146949

**Published:** 2016-02-12

**Authors:** Bastian O. Sabel, Kristijan Buric, Nora Karara, Kolja M. Thierfelder, Julien Dinkel, Wieland H. Sommer, Felix G. Meinel

**Affiliations:** Institute for Clinical Radiology, Ludwig-Maximilians-University Hospital, Munich, Germany; The University of Chicago, UNITED STATES

## Abstract

**Objectives:**

To investigate the feasibility of high-pitch CT pulmonary angiography (CTPA) in 3^rd^ generation dual-source CT (DSCT) in unselected patients.

**Methods:**

Forty-seven patients with suspected pulmonary embolism underwent high-pitch CTPA on a 3^rd^ generation dual-source CT scanner. CT dose index (CTDI_vol_) and dose length product (DLP) were obtained. Objective image quality was analyzed by calculating signal-to-noise-ratio (SNR) and contrast-to-noise ratio (CNR). Subjective image quality on the central, lobar, segmental and subsegmental level was rated by two experienced radiologists.

**Results:**

Median CTDI was 8.1 mGy and median DLP was 274 mGy*cm. Median SNR was 32.9 in the central and 31.9 in the segmental pulmonary arteries. CNR was 29.2 in the central and 28.2 in the segmental pulmonary arteries. Median image quality was “excellent” in central and lobar arteries and “good” in subsegmental arteries according to both readers. Segmental arteries varied between “excellent” and “good”. Image quality was non-diagnostic in one case (2%), beginning in the lobar arteries. Thirteen patients (28%) showed minor motion artifacts.

**Conclusions:**

In third-generation dual-source CT, high-pitch CTPA is feasible for unselected patients. It yields excellent image quality with minimal motion artifacts. However, compared to standard-pitch cohorts, no distinct decrease in radiation dose was observed.

## Introduction

Due to its high sensitivity and specificity [1,2] its wide availability and its potential to assess mediastinal and parenchymal structures for alternate diagnoses, computed tomography pulmonary angiography (CTPA) has become a preferred diagnostic imaging method within the diagnostic algorithm for suspected PE. [2]

Dual-source CT scanners provide high-pitch dual source protocols with pitch factors up to 3.4, allowing continuous volume coverage of the whole thorax in less than one second. The major advantages of this fast acquisition lie in a significant reduction of motion artifacts, [3] but it has also been suggested to lower radiation exposure of the patient. [4–6]

On the other hand, these advantages of high-pitch acquisition protocols are traded in for increased image noise in heavier patients. This is due to limitations in tube current. Since X-ray tubes are limited in their maximum current, only a certain amount of radiation can be emitted during the extremely short acquisition times of high-pitch protocols. This is often not sufficient to produce diagnostic image quality in large patients. Until recently it was therefore not possible to apply high-pitch protocols in all patients without running the risk of affecting the diagnostic value of the images. Published studies on high-pitch acquisition protocols for CTPA have been performed in selected patient populations with relatively low body weight. [4–6]

Recently, a third-generation of dual-source CT systems has been developed with substantially increased maximum tube current [7]. We hypothesized that the higher tube current of the 3^rd^ generation dual source CT scanners renders high-pitch CTPA protocols feasible for unselected patients.

## Material and Methods

### Patient selection and study design

This study was conducted as a retrospective single-center study. The study protocol was approved by the responsible institutional review board (Ethikkommission of the Ludwig-Maximilians-University, Munich) with waiver of informed consent and performed in accordance with the Declaration of Helsinki. In total, the study comprised 47 patients who had been referred to our department for a clinically indicated CTPA to exclude acute PE between March and July 2014.

### CT acquisition protocol

CT acquisition parameters are summarized in Table 1. The patients were examined on the third-generation dual-source CT system (SOMATOM Force, Siemens Healthcare, Forchheim, Germany) [7] in dual-source, helical acquisition mode at a gantry rotation time of 0.25 s / rotation, pitch 1.9, 0.75 mm section thickness and 192 x 0.6 mm detector rows. In all examinations, automated attenuation-based tube voltage selection (CAREkV, Siemens Healthcare) [8] and tube current modulation along the z axis (CAREDose, Siemens Healthcare) was used with a quality reference tube voltage of 100 kV and a quality reference tube current time product of 220 mAs. The scan range covered the entire pulmonary parenchyma, extending from the costophrenic angle to the level of the pulmonary apex. All patients were asked to hold their breath in inspiration during the examination. Fifty mL of intravenous contrast agent were injected at a flow rate of 5mL/s, followed by 100 mL of saline injected at the same flow rate. A bolus triggering algorithm was used, which automatically started the scan 6 seconds after a prespecified threshold of 100 HU was reached in the main pulmonary artery.

**Table 1:** Acquisition Parameters. Acquisition parameters. Data is shown as median (range) where appropriate.

Acquisition Parameters	
**CT system**	Siemens Somatom Force
**Quality reference tube voltage**	100 kV
**Selected kV, range**	100 (80–130) kV
**Quality reference tube current time product**	220 mAs
**Selected effective tube current time product, range**	236 (134–328) mAs
**Rotation Time**	0.25 s/rot
**Detector rows**	192 x 0.6 mm
**Pitch**	1.9

### Radiation metrics

The volume CT dose indices (CTDI_vol_) as well as the dose length products (DLP) were retrieved from the dose report stored in the picture archiving and communication system (PACS, Syngo Imaging 2010, Siemens Healthcare). Effective radiation doses were estimated by multiplying the DLP with a standard conversion factor for adult chest CT of 0.0146 mSv/mGy*cm. [9] Anteroposterior (AP) and lateral chest diameters (LAT) were measured on topograms at the level of the carina. Based on the effective diameter (ED) of the chest ($ED=\sqrt{\left(AP\cdot LAT\right)}$), [10] size-specific dose estimates (SSDE) were calculated using the size- specific conversion factor f_size_ of the AAPM Report 204 (*SSDE* = *fsize* ⋅ *CTDIvol*). [11]

### Image reconstruction

The image series were reconstructed with a slice thickness of 2 mm and 2 mm increment. For reconstructions, a medium sharp vascular Bv36d kernel was used. An intermediate iterative reconstruction strength of 3 was used for all reconstructions.

### Analysis of objective image quality and dose efficiency

Quantitative analysis of image quality was performed to determine image noise, signal-to-noise ratio (SNR) and contrast-to-noise ratio (CNR). For each patient, regions of interest (ROIs) were placed on an axial slice in the lumen of the main pulmonary artery, the right pulmonary artery, the left pulmonary artery, one segmental pulmonary artery on each side and in the paraspinal muscles. The paraspinal muscle was used as reference because it provides homogeneous attenuation with almost no contrast enhancement in the pulmonary arterial phase. [4] Image noise was defined as the standard deviation of the CT attenuation in the main pulmonary artery. Contrast-to-noise-ratio in the central pulmonary arteries (CNRcpa) was calculated as ($CNRcpa=\frac{CTApa-CTAm}{IN}$). Contrast-to-noise-ratio in the segmental pulmonary arteries (CNRspa) was accordingly calculated as ($CNRspa=\frac{CTAspa-CTAm}{IN}$). Signal-to-noise-ratio in the central pulmonary arteries (SNRcpa) was calculated as ($SNRcpa=\frac{CTApa}{IN}$). Signal-to-noise-ratio (SNR) in the segmental pulmonary arteries (SNRspa) was accordingly calculated as ($SNRspa=\frac{CTAspa}{IN}$).

### Subjective assessment of diagnostic confidence and motion artifacts

The analysis of image quality was performed independently by two radiologists (3 and 5 years of experience in chest CT respectively), in random order and blinded to each other’s evaluation results. Both observers had access to all axial source images, coronal and sagittal reformation images and were allowed to freely adjust window settings.

The subjective overall image quality was scored on a 5-point scale as follows for the central, lobar, segmental and subsegmental pulmonary arteries respectively: 5 = excellent, optimal enhancement to allow unambiguous diagnosis of the presence or absence of a clot; 4 = good, clear enhancement of the artery with slight blurring; 3 = sufficient, reduced enhancement of the artery which still allows confident diagnosis of the presence or absence of a clot; 2 = poor, inadequate opacification of substantial blurring causing substantial uncertainty regarding the presence or absence of a clot; 1 = non-diagnostic.

Motion artifacts affecting the pulmonary vasculature on CT images (including cardiac motion and breathing artifacts) were rated as "none", "minor" or "major". Minor motion artifacts were defined as not relevant for diagnostic confidence, whereas major artifacts were defined as substantial, hampering the diagnostic evaluation of the affected areas. Motion artifacts were independently rated by the two readers.

## Results

### Patient Characteristics

Patient characteristics are summarized in Table 2. Forty-seven consecutive patients (20 men and 27 women aged between 38 and 92, median age: 66 years) with suspected pulmonary embolism were analyzed. Pulmonary embolism was confirmed in 11 patients. The median effective chest diameter was 28 cm (interquartile range 26–30 cm), the median BMI was 25 (interquartile range 23–28).

**Table 2:** Patients Demographics. Composition of the study population. Data is shown as median (range) unless marked otherwise.

Parameters	All patients
**No. Patients**	47
**Age, y**	66 (32–94)
**Male, n (%)**	20 (43)
**Effective chest diameter, cm**	28 (22–37)
**Body Weight, kg**	73 (50–121)
**BMI**	25.0 (20.5–42.0)

### Radiation dose

The median tube voltage, which was automatically selected by the attenuation based tube voltage selection algorithm, was 100 kV, ranging from 80 kV to 130 kV. Median CTDI_vol_ was 8.1 mGy and median DLP was 274.1 mGy*cm. Median effective dose was 4.0 mSv and median SSDE was 18.5 mGy.

### Objective image quality and dose efficiency

The median CNR was 29.2 in the central pulmonary arteries and 28.2 in the segmental pulmonary arteries. For SNR, the median in the central pulmonary arteries was measured as 32.9 and as 31.9 in the segmental pulmonary arteries.

### Subjective image quality

**Fig 1:**
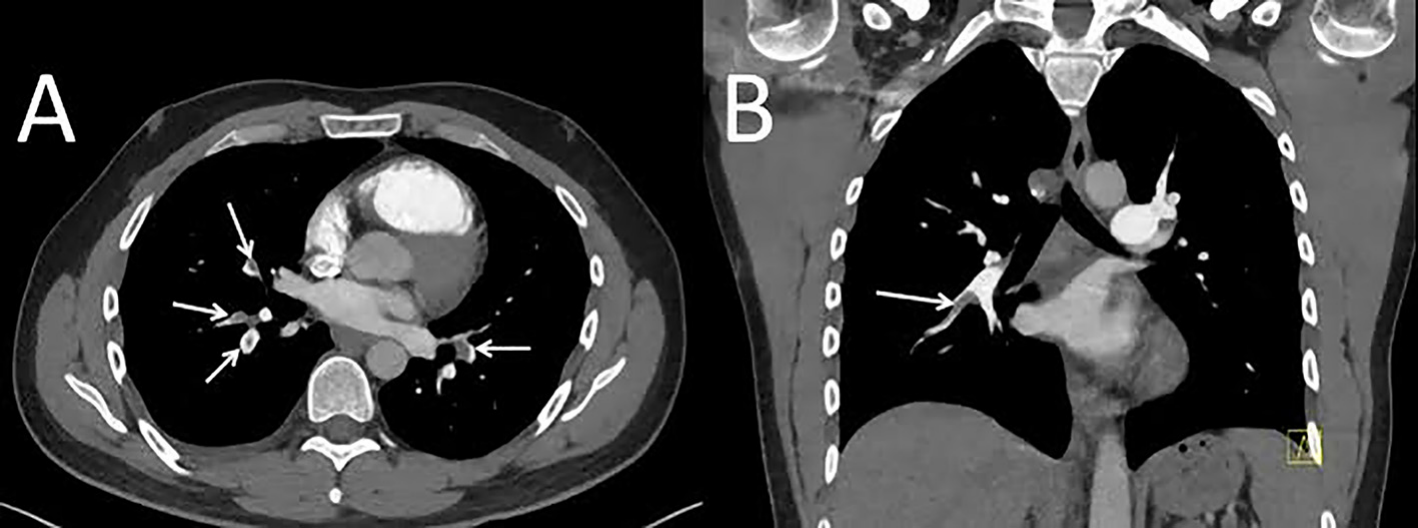
High-pitch CT pulmonary angiogram shown as transverse (A) and coronal (B) reconstructions in a 37-year old normal-weight male patient (effective chest diameter 29 cm) with acute bilateral pulmonary embolism (arrows). Image quality was rated as "excellent" by both readers.

**Fig 2:**
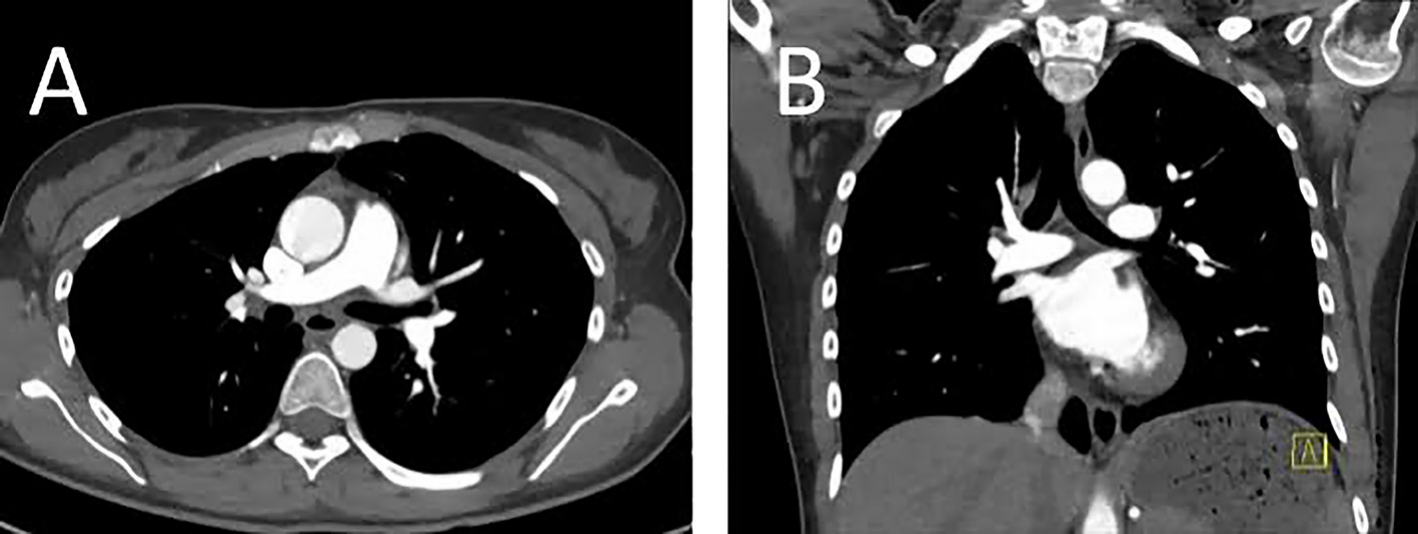
High-pitch CT pulmonary angiogram shown as transverse (A) and coronal (B) reconstructions in a 33-year old lean female patient (BMI 21, effective chest diameter 23 cm) with no evidence of pulmonary embolism. Image quality was rated as "excellent" by both readers.

The results of the evaluation of the subjective image quality are summarized in Table 3. Median image quality was “excellent” (e.g. Fig 1 and Fig 2) in central and lobar arteries and “good” in subsegmental arteries according to both readers. Segmental arteries varied between “excellent” and “good”. Image quality was derogated by incomplete mixing of contrast media with non-opacified blood in the pulmonary vasculature in two cases. The scan was non-diagnostic in one patient (2%) with an effective chest diameter of 31,6 cm, beginning in the lobar arteries. This scan was performed too early so that no sufficient enhancement of the vascularization was achieved. The SNR was 13.9 in the central and 10.6 in the segmental pulmonary arteries. CNR was measured with 11.9 in the central and 8.6 in the segmental pulmonary arteries.

**Table 3:** Image Quality. The subjective overall image quality was rated as excellent, good, poor, sufficient and non-diagnostic for the central, lobar, segmental and subsegmental pulmonary arteries respectively. Results are given as absolute frequencies for both readers.

	Reader 1		Reader 2	
**Central pulmonary arteries**	** **			** **
excellent	30			32
Good	14			12
Sufficient	1			1
Poor	2			2
Non diagnostic	0			0
**Lobar pulmonary arteries**				
excellent	27			23
Good	15			15
Sufficient	3			7
Poor	1			1
Non diagnostic	1			1
**Segmental pulmonary arteries**				
excellent	21			26
Good	18			15
Sufficient	7			4
Poor	0			1
Non diagnostic	1			1
**Subsegmental pulmonary arteries**				
excellent	18			18
Good	15			20
Sufficient	11			8
Poor	2			1
Non diagnostic	1			0

### Motion artifacts

There was no case of major artifacts. The distribution of motion artifact ratings for both readers was as follows: Minor motion artifacts were reported in 12 cases by reader 1 while 11 cases were noted by reader 2. The reports overlapped in 10 of the cases. The remaining cases, 35 and 36 respectively, represented no motion artifacts.

### Influence of patient size on image quality

**Fig 3:**
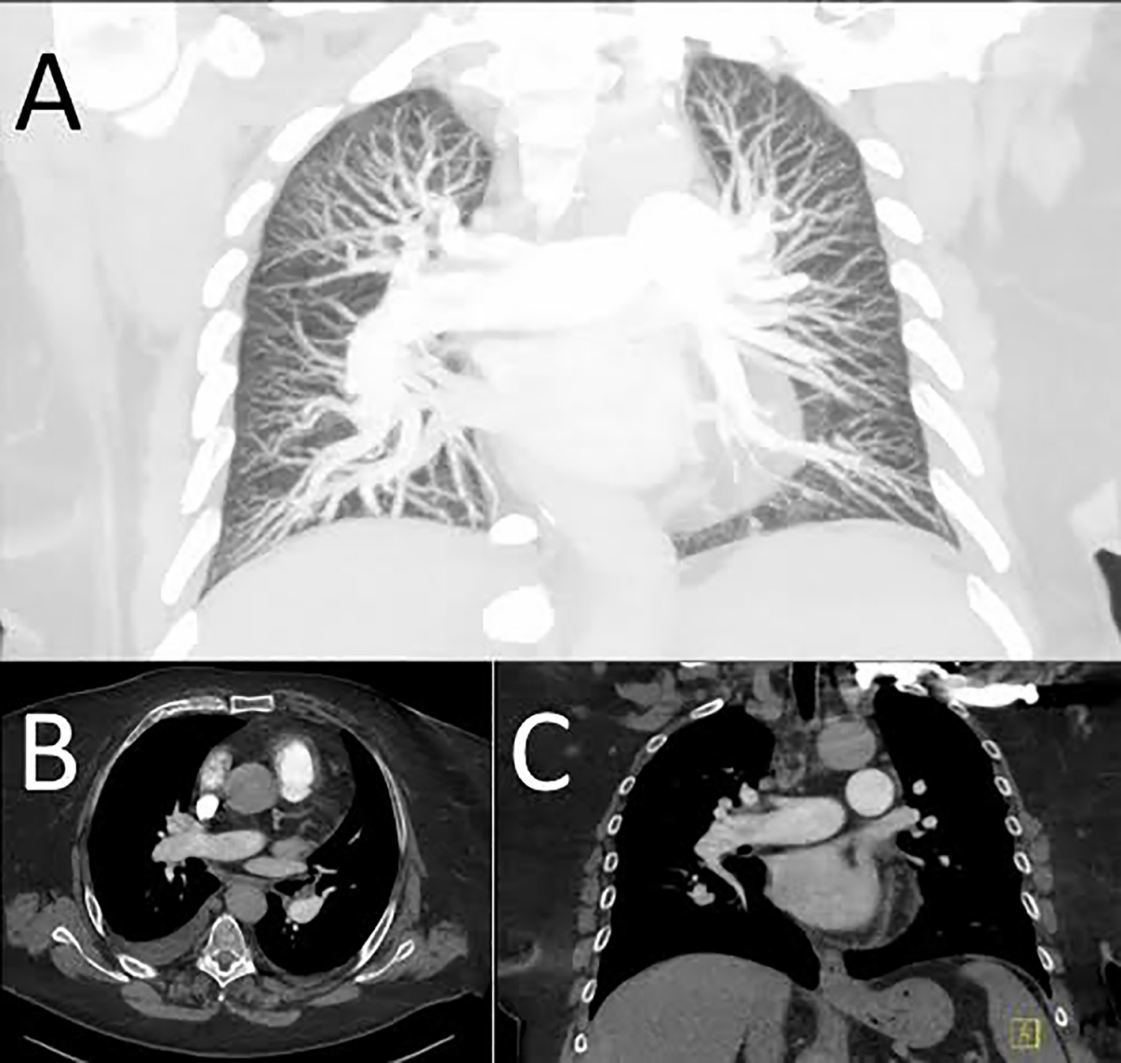
High-pitch CT pulmonary angiogram shown as coronal maximum intensity projection (A), as well as transverse (B) and coronal (C) multiplanar reformats in a 63-year old obese female patient (effective chest diameter 37.3 cm) with no evidence of pulmonary embolism. Image quality was rated as "good" on the central and lobar arteries and “sufficient” in the segmental arteries by both readers.

In order to evaluate the influence of patient size on image quality, patients with an effective chest parameter above the median of the whole cohort (27.7 cm) were compared with those below that threshold. Table 4 summarizes the results of the comparison of both groups. CTDI, DLP, effective dose and SSDE were significantly higher in patients with a wider effective chest diameter. Also, patients with a wider effective chest diameter showed lower intravascular enhancement as well as lower subjective and objective image quality on all levels. Nevertheless, median image quality was still "good" on all levels even in the group of larger patients (Fig 3). There was no difference in motion artifacts between both groups.

**Table 4:** Influence of Patient Size on Image Quality. Patients with an effective chest parameter above the median of the whole cohort (27.7 cm) were compared with those ranging below that threshold. Data is shown as median (interquartile range).

	Eff. chest diameter < 27.7	Eff. chest diameter > 27.7	P-value
kV	90 (80–130)	110 (90–130)	< 0.001
DLP	226 (205–259)	375 (295–505)	< 0.001
effective dose	3.3 (3.0–3.8)	5.5 (4.3–7.4)	< 0.001
SSDE	16.2 (14.7–17.6)	23.8 (19.8–36.6)	< 0.001
attenuation right pulmonary artery	526 (440–654)	372 (323–477)	0.006
attenuation left pulmonary artery	557 (435–663)	379 (310–468)	0.014
attenuation segmental arteries right	513 (420–625)	351 (266–419)	0.003
attenuation segmental ateries left	494 (411–622)	340 (267–419)	0.005
SD MPA	14.4 (12.4–16.4)	12.4 (10.7–15.0)	0.083
SNR central	41.1 (31.0–26.7)	30.2 (25.4–35.2)	0.132
CNR central	36.8 (26.7–41.3)	26.3 (20.7–30.1)	0.088
SNR segmental	37.9 (30.2–44.9)	25.8 (21.9–34.5)	0.069
CNR segmental	33.6 (26.5–41.0)	22.2 (17.2–29.9)	0.043
Image quality central	5.0 (5.0–5.0)	4.0 (4.0–5.0)	< 0.001
Image quality lobar arteries	5.0 (4.0–5.0)	4.0 (4.0–5.0)	0.002
Image quality segmental arteries	5.0 (4.0–5.0)	4.0 (4.0–5.0)	0.003
Image quality subsegmental arteries	4.5 (4.0–5.0)	4.0 (3.0–4.0)	0.002
Motion artifacts	0.0 (0.0–0.8)	0.0 (0.0–0.0)	0.771

## Discussion

This pilot study evaluated the feasibility, image quality and radiation dose of high-pitch CT pulmonary angiography in an unselected population using third-generation dual source CT. Since image noise increases with patient size, the maximum patient weight for which high-pitch protocols can be performed is determined by the maximum available tube current that can be delivered by the respective CT scanner. [12,13] In the past, only relatively slim patients have been able to benefit from this technique, as the CT scanner had already reached the maximum tube output. [12] We hypothesized that this limitation could be overcome by using the newly developed third-generation dual-source CT scanner with substantially raised tube current capacities. [7]

The results of our study show that this technique reliably yields excellent subjective image quality for depicting PE in the central and lobar arteries. There was also excellent to good image quality down to the subsegmental arteries. This observation is well supported by the determined parameters of the objective image quality. Median CNR was 29.2 in the central arteries and 28.2 in the segmental arteries. According to the literature, a minimum CNR of only five is required for a reliable detection of pulmonal emboli. [14] Adequate vessel enhancement has shown to be crucial for the detection of a pulmonary embolism. [15,16] It has been reported that a minimum radiodensity value of 250 HU permits confident exclusion of an embolus. [16–20] The median of the opacification in the pulmonal arteries in our study exceeded this value on every level beginning at 421 HU in the segmental arteries. In 96% of the examined patients, all opacification measurements were above the 250 HU threshold. The use of high-pitch CTPA thus led to optimal vessel enhancement in the majority of patients.

As the high-pitch DSCT allows a shortening of the acquisition time and reduces data overlap, a significant dose reduction was expected. [3,4,6] However, compared to standard-pitch cohorts with state-of-the art CT equipment reported in the literature, no distinct decrease in radiation dose was observed. For example, Tacelli and colleagues reported mean DLP values of 215.41 mGy * cm, which is somewhat lower than the median DLP of 274 mGy * cm found in our study. This is in contrast to other studies, which had shown dose reduction in high-pitch protocols. [4–6] We conclude that according to our data high-pitch acquisition *per se* does not substantially reduce radiation dose. Rather, the dose reduction observed in other high-pitch cohorts was mainly a result of tube current limitations. With an image quality much higher than required for a reliable assessment of PE, it appears that there is substantial room for dose optimization in our cohort. The quality reference settings for tube voltage and/or tube current can safely be decreased, such that image quality will be lower but still diagnostic.

Another widely accepted and effective method of reducing radiation dose is lowering the tube voltage based on [15,21,22]. By lowering the setting from 120 to 80 kV, an average dose reduction from 40–60% can be achieved. [23,24] To date, this strategy had been restricted to slim patients, as reducing the tube voltage sensibly increases image noise, leading to deterioration of image quality in larger patients. [25,26] Lowering the kilo voltage is especially attractive for CT angiographic applications. Low kV examinations maximize the photoelectric effect, as in this case, the applied voltage is closer to the k-edge of iodine (33.2 keV). As a consequence, this leads to increased contrast-to-noise ratio and improved vascular enhancement. [25,27–29] In our study, we used an automated tube voltage selection algorithm to select the most dose-efficient combination of tube voltage and tube current for each individual patient. [30]

Besides poor contrast enhancement, motion artifacts are indicated to be the major causes for obscuring the presence of PE in pulmonary angiograms. [16] Our observations confirm the significant reduction of motion artifacts found in a previous study on high-pitch CTPA.[31] The increased pitch is rendered feasible by the simultaneous use of both tubes and detectors, providing overlapping projection data from both systems and avoiding undersampling artifacts. As a result of the high-pitch acquisition in combination with a short gantry rotation, motion artifacts are much less likely to occur, which is particularly beneficial in patients who are unable to hold their breath. As an additional benefit not examined in our study, high-pitch protocols also reduce cardiac motion artifacts and thus improve assessment of the cardiovascular structures as well [32].

The comparison of patients with an effective chest parameter below the median to those with an effective chest parameter above the median showed lower intravascular attenuation and higher image noise in the more corpulent patients compared to the leaner patients. The lower intravascular attenuation can in part be explained by a selection of higher kV values in larger patients. Subjective and objective image quality were superior in leaner patients. As expected, the administered radiation dose rises with the chest diameter. However, even in the cohort with the higher effective chest diameter, all patients featured good to excellent image quality. Therefore, no restrictions regarding patient’s weight have to be made when applying high-pitch CTPA in a third generation CT scanner.

The introduction of high-pitch protocols with shorter acquisition times drives the development of optimized protocols in order to obtain uniform and consistent vascular enhancement in the pulmonary arteries. [33] One observation made during this study was that incomplete mixing of contrast media with non-opacified blood in the pulmonary vasculature did occasionally occur. This might be caused by the faster start of the scan and the shorter acquisition times in third-generation scanners. Especially when using small volume of iodinated contrast material, accurate delay time is crucial for excellent opacification of the pulmonary vasculature. [4] Further investigation needs to be carried out, but a prolongation of the delay time may be a reasonable solution for this issue.

The results of this study should be interpreted in the context of the study design and its limitations. First, the analysis was directed towards quantitative and qualitative image criteria and did not evaluate the sensitivity or specificity of PE identification compared with a reference standard. Second, measurements of SNR and CNR were carried out only in the central and segmental pulmonary arteries. Therefore, decreased values in the peripheral vessels might have been missed. This decision was made to avoid the considerable risk of including misleading partial volumes in the measurements. Finally, third-generation high-pitch DSCT scanners are not widely available, which may limit the widespread application of this technique.

The impact of the administered volume of contrast media was not the focus of our study. Bolen et al found that high-pitch protocols require amounts of contrast media similar to non high-pitch examinations. [34] In our cohort we observed a contrast enhancement with a median of over 420 HU in the segmental pulmonary arteries with low KeV values selected by the CAREkv protocol. Therefore, there should be some room for further contrast media reduction. On the other hand, the CTPA protocol is designed for an emergency room setting and therefore needs to be robust and ensure a doubtless exclusion of pulmonary embolism.

As a conclusion of our study, high-pitch CTPA is feasible even for unselected patients due to the increased tube current capacities of third-generation dual-source CT system. This technique yields excellent image quality with minimal motion artifacts. However, compared to standard-pitch cohorts reported in literature, no distinct decrease in radiation dose was observed. Based on our pilot study, high-pitch CTPA can safely be used for unselected patients at third-generation dual-source CT, but quality reference settings for tube voltage and/or tube current should be further decreased to optimize radiation dose.

## Key points

1. With third-generation dual-source CT, high-pitch CTPA is feasible in unselected patients.
2. This approach yields excellent image quality with minimal motion artifacts.
3. Reference settings for tube potential and current should be optimized for dose-saving.

## Abbreviations

CNR: contrast-to-noise-ratio
CT: computed tomography
CTAm: average of CT attenuation in the paraspinal muscle
CTApa: average of CT attenuation in the pulmonary artery
CTAspa: average of the CT attenuation in one right and left segmental pulmonary artery
CTDI_vol_: volumetric CT dose index
DLP: dose length product
DSCT: dual-source CT system
ED: effective dose
HU: Hounsfield unit
IN: image noise inside the main pulmonary artery
PE: pulmonary embolism
ROI: region of interest
SSDE: size-specific dose estimate
